# Phenolics of Maqui Leaf Residues Exhibit Antioxidant Properties Against Ozone-Induced Oxidation in Fish Model Systems

**DOI:** 10.3390/antiox14030263

**Published:** 2025-02-26

**Authors:** Miguel Angel Varas Condori, María Fernanda Arias-Santé, Raquel Bridi, Miguel Ángel Rincón-Cervera, Omar Porras, Angélica Reyes-Jara, Adriano Costa de Camargo

**Affiliations:** 1 Nutrition and Food Technology Institute, University of Chile, Santiago 7830490, Chile; mvaras@inta.uchile.cl (M.A.V.C.); ma.fernanda.arias@inta.uchile.cl (M.F.A.-S.); marincer@inta.uchile.cl (M.Á.R.-C.); omar.porras@inta.uchile.cl (O.P.); areyes@inta.uchile.cl (A.R.-J.); 2 Departamento de Química Farmacológica y Toxicológica, Facultad de Ciencias Químicas y Farmacéuticas, Universidad de Chile, Santiago 8380000, Chile; raquelbridi@ciq.uchile.cl; 3 Food Technology Division, University of Almería, 04120 Almería, Spain

**Keywords:** *Aristotelia chilensis*, optimization, natural antioxidants, lipid oxidation, salmon

## Abstract

Growing concern about food quality and safety has driven the search for natural food additives. Furthermore, maqui leaf residue (MLR), recovered from infusions that have long been part of traditional Chilean herbal medicine, still conserves a high content of phenolic compounds. The aim of this study was to optimize the ultrasound-assisted extraction (UAE) of phenolics from MLR and evaluate their potential effect on salmon preservation. The optimized parameters for UAE (6 min, MLR:Water = 1:30, and 70 W), obtained by using a Box–Behnken design, rendered the highest total phenolic content and antioxidant capacity. Furthermore, UAE showed a higher concentration of phenolic compounds compared to conventional extraction techniques, as evaluated by UPLC-ESI-MS/MS. The salmon with MLR had up to four times lower levels of thiobarbituric acid reactive substances, induced by ozonated water treatment, than the sample without this natural antioxidant. Moreover, MLR exhibited similar or superior effectiveness compared to synthetic antioxidants, such as butylated hydroxyanisole (BHA), 3,5-Di-tert-4butylhydroxytoluene (BHT). This study highlights the use of short-time aqueous ultrasound extraction as an environmentally friendly technology that allows the recovery of phenolic compounds from MLR, with potential application as a natural preservative that may replace or decrease the use of BHA and/or BHT.

## 1. Introduction

*Aristotelia chilensis* (Molina) Stuntz (Elaeocarpaceae), commonly known as maqui, is an evergreen tree or shrub that grows in Chile and western Argentina [1]. Maqui leaves are used to prepare infusions that are recognized as part of traditional Chilean herbal medicine [2]. Its leaves have long been used in traditional medicine to prepare infusions due to their potential health benefits [2]. These infusions may possess astringent, antidiarrheal, antioxidant, and anti-inflammatory properties. They also have been used for treating kidney pain, stomach ulcers, various digestive ailments, fever, and wound healing, and may present cardioprotective activity [3,4]. These properties may be attributed at least in part to their phenolic bioactives.

Phenolic compounds are found in the plant matrix, primarily within the vacuoles of plant cells [5]. Various methods are used to extract them, with solid–liquid extraction being one of the most common. This technique typically involves organic solvents such as methanol, ethanol, chloroform, ethyl acetate, n-hexane, propanol, and acetone [6], to name a few, as well as their mixtures with water. However, the use of organic solvents has several drawbacks, including the difficulty of removing impurities and the potential toxicity of some solvents, which limits their application in food. Additionally, their use is associated with negative environmental impacts [7].

Emerging technologies have been developed to reduce or eliminate the use of organic solvents in polyphenol extraction, minimizing their environmental impact [7,8]. Among these, ultrasound-assisted extraction (UAE) has gained attention as a sustainable alternative. This technique generates pressure waves that create cavitation bubbles, which collapse and release high amounts of energy, enhancing the extraction process [9]. UAE has been successfully applied to extract phenolic compounds from tea leaves, demonstrating its ability to shorten extraction times and improve yield [7].

As for the safety of food, ozone has been widely used in meat preservation due to its strong oxidizing properties, which effectively inactivate a broad range of pathogenic and spoilage microorganisms, including Gram-positive and Gram-negative bacteria, as well as spores and vegetative cells [10,11]. However, ozone can also react with polyunsaturated fatty acids, triggering lipid peroxidation that leads to the formation of ozonides, which subsequently decompose into lipid peroxides [12]. This effect was demonstrated in tilapia [13], where immersing filets in ozonated water reduced the total mesophilic count. However, a slight increase in lipid oxidation was also observed. Similar findings have been reported for tilapia [14] and salmon [15]. Salmon is widely used as a model to evaluate the antioxidant effectiveness of phenolic extracts [16,17,18], mainly due to its high content of polyunsaturated fatty acids, which makes it particularly susceptible to lipid oxidation [19].

In summary, one of the most studied properties of maqui leaves is their antioxidant capacity [20,21,22], but there are no reports on leaf residues obtained after an infusion process. Compared to other native Chilean fruit leaves, it has been reported that the ethanolic extract of maqui leaves has a higher phenolic content and antioxidant capacity than murta (*Ugni molinae* Turcz.) and its leaves [20]. Considering this literature gap, the problems related to the use of organic solvents, and the efficient use of time and energy, the aim of this study was to optimize the obtention of an aqueous food-grade phenolic extract. To achieve this goal, an ultrasound-assisted procedure to recover a high level of natural antioxidants from maqui leaf residue (MLR), which does not contemplate the use of organic solvents and reduces the extraction time, was developed by using a response surface methodology with a Box–Behnken design. Its potential application as a natural antioxidant additive in fish products was also addressed using an ozone-induced lipid oxidation salmon model, highlighting its potential as an alternative to synthetic antioxidants like BHA and BHT.

## 2. Materials and Methods

### 2.1. Plant Material and Sample Preparation

Commercial maqui leaves (*Aristotelia chilensis*) from a local company were used. Damaged leaves were removed, and the selected ones were stored (−4 °C) in plastic bags until processing. Maqui leaf infusions (4% *w*/*v*) were prepared at 80 °C for 5 min in a thermostatic water bath (LSB-130S, LabTech, Sorisole, Bergamo, Italy). The infusion was separated from MLR by vacuum filtration. The residue was dried in an oven (Vacucell EC 111, MMM Group, Planegg, Múnich, Germany) at 40 °C for 48 h, and then crushed, sieved, and stored at −4 °C until further processing.

### 2.2. Chemicals and Reagents

Methanol, acetone, acetic acid (glacial) 100%, 2-thiobarbituric acid (TBA), hydrochloric acid ACS (37%), 2,4,6-Tris(2-pyridyl)-s-triazine (TPTZ), butylated hydroxyanisole (BHA), and 3,5-Di-tert-4butylhydroxytoluene (BHT) were purchased from Merck (Darmstad, Germany). Iron (III) chloride (97%), sodium acetate anhydrous (≥99%), sodium phosphate monobasic monohydrate ACS (≥98%), sodium phosphate dibasic ACS (≥99%), sodium carbonate ACS (≥99.5%), Folin and Ciocalteu’s phenol reagent 2 N, trichloroacetic acid (TCA) ACS (≥99%), 1,1,3,3-Tetraethoxypropane (≥96%), gallic acid, flourescein sodium salt, (±)-6-Hydroxy-2,5,7,8-tetramethylchromane-2-carboxylic acid (Trolox), 2,2′-azobis(2-methyl-propionamidine) dihydrochloride (AAPH), phenolic acids (gallic, syringic, ferulic, chlorogenic, caffeic, and p-coumaric), and flavonoids (catechin, rutin, quercetin, luteolin, kaempferol, epicatechin, myricetin, and isorhamnetin) were purchased from Sigma-Aldrich (St. Louis, MO, USA)

### 2.3. Conventional Extraction of Polyphenols

The extraction of polyphenols from maqui leaves and MLR was performed using a solvent mixture of methanol–water (3:4:3 *v*/*v*/*v*) in a 1:10 ratio (*w*/*v*). The extraction was carried out in a thermostatic water bath with continuous stirring (50 RPM) for 10 min at 30 °C. After extraction, the supernatant was separated from the maqui leaves by vacuum filtration. The choice of solvent mixture was previously optimized through a mixture design, based on maximizing the extraction of total phenolic content (Table S1, Figure S1).

### 2.4. Optimization of Ultrasound-Assisted Polyphenol Extraction

An ultrasonic homogenizer (Sonic VCX 750, Sonics & Materials INC, Newtown, CT, USA) with a frequency of 20 kHz was used (Figure S2). The sample was placed in a beaker with water, and a standard 1/2” diameter probe was introduced to provide the necessary power according to the optimization design. The pulse of the ultrasonic homogenizer (on 20 s, off 5 s) remained constant. The optimization was carried out using a response surface methodology with a Box–Behnken design. Three independent variables with two levels were considered according to the steps taken in previous studies [23,24]. The conditions were A, time (1–30 min); B, MLR–water (1:10–1:40); and C, power (30–110 W). The response variables were total phenolic content (TPC) and antioxidant capacity (FRAP and ORAC).

Multiple regression analysis was performed to obtain prediction models for each response variable. Determination coefficients (R^2^ and adjusted R^2^) and lack of fit were used to assess the model’s fit to the experimental data. The optimal parameters for the extraction of phenolics from MLW were determined using a desirability function based on the highest values of TPC, FRAP, and ORAC. Model validation was performed by comparing the predicted results with the experimental values.

Additional extractions with water (without ultrasound) and with a methanol–water–acetone (3:4:3 *v*/*v*/*v*) mix, using the same MLW–water ratio and extraction time as the optimized extract conditions, were carried out for comparison purposes.

### 2.5. Total Phenolic Content

Total phenolic content (TPC) was determined according to the literature [25]. Briefly, 150 µL of the samples was mixed with 2000 µL of Folin–Ciocalteu reagent (diluted 1/10 *v*/*v* with distilled water), 400 µL of sodium carbonate (20% *w*/*v*), and 450 µL of distilled water. The samples were incubated for 30 min, and absorbance was read at 765 nm using a spectrophotometer (N4S UV-VIS, Hinotek, Ningbo, Zhejiang, China). The results were expressed as mg gallic acid equivalents (GAE)/g.

### 2.6. Ferric Reducing Antioxidant Potential (FRAP)

FRAP was determined following the methodology of Benzie and Strain [26]. Briefly, 300 µL of the samples was reacted with 2700 µL of FRAP solution (TPTZ–FeCl_3_–acetate buffer, 1:1:10), incubated for 30 min at 37 °C, and protected from light, and absorbance was measured at 593 nm using a spectrophotometer (N4S UV-VIS, Hinotek, Ningbo, Zhejiang, China). The results were expressed as µmol Trolox equivalents (TE)/g.

### 2.7. Oxygen Radical Absorbance Capacity (ORAC)

ORAC was determined according to the methodology reported by Toledo-Merma et al. [25] with modifications. Briefly, 175 µL of phosphate buffer 75 mM (pH 7.4), 30 µL of fluorescein 5.83 × 10⁻^7^ M, and 20 µL of sample were reacted in a 96-well black plate and incubated for 20 min at 37 °C while being protected from light. Then, 25 µL of AAPH 0.1 M was added, and fluorescence readings (λ excitation = 480 nm, λ emission = 510 nm) were taken every 2 min using a microplate reader (Infinite M200 Pro, TECAN, Männedorf, Zúrich, Switzerland) for 2 h and 20 min at 37 °C. The area under the curve was calculated at 80% fluorescence decay. The results were expressed as µmol TE/g.

### 2.8. Phenolic Profile (UPLC-ESI-MS/MS)

The phenolic profile was analyzed following the methodology described by de Camargo et al. [27] using ultra-performance liquid chromatography coupled with electrospray ionization tandem mass spectrometry (UPLC-ESI-MS/MS). The extracts were filtered through a Millex-LCR hydrophilic PTFE membrane (0.45 µm pore size) and stored in vials at −20 °C for further analysis. An ABSciex triple Quad 4500 mass spectrometer supplied with an electrospray (TurboV) interface combined with an Eksigent Ekspert Ultra LC100 with an Ekspert Ultra LC100-XL autosampler system (AB/Sciex, Concord, ON, Canada) was used. A UPLC LiChrospher 100 RP-18 end-capped column (125 mm × 4 mm i. d., 5 μm; Merck KGaA, Darmstadt, Germany) was applied in chromatographic separation. Then, 0.1% formic acid (mobile phase A) and methanol (mobile phase B) were used for gradient elution as follows: 0–1 min, 5% B; 1–12 min, 5–50% B; 12–13 min, 50–50% B; 13–14 min, 50–5% B; and 14–15 min, 5% B. The flow rate was 0.5 mL/min, the injection volume was 10 μL, and the column temperature was 40 °C. Electrospray was employed in the negative mode and the following parameters were employed: curtain gas = 30 psi; collision gas = 10 psi; ion spray voltage = −4500 V; temperature = 650 °C; ion source gas 1 = 50 psi; ion source gas 2 = 50 psi; and entrance potential = 10 V. Phenolic acid standards (gallic, syringic, ferulic, chlorogenic, caffeic, and p-coumaric acids) and flavonoid standards (catechin, rutin, quercetin, luteolin, kaempferol, epicatechin, myricetin, and isorhamnetin) were used for identification and quantification purposes. The limits of detection (LOD), quantification (LOQ), and R^2^ values for each polyphenol are shown in Table S2.

### 2.9. Evaluation of MLR on Lipid Oxidation in a Fish Model System

#### 2.9.1. Salmon Oxidation with Ozone

Ozonized water was used as the oxidation agent for salmon. A system consisting of an ozone generator (O&L 3.0 RM, Ozone&Life, São José dos Campos, São Paulo, Brazil), ozonation tower (Ozone&Life, São José dos Campos, São Paulo, Brazil), ozone monitor (106-H, 2B Tech, Broomfield, CO, USA), and a thermal catalyst (Ozone&Life, São José dos Campos, São Paulo, Brazil) was used. Then, 250 mL of water was placed in the ozonation tower and subjected to a flow of 37 µg/mL for 10 min.

#### 2.9.2. Storage Test

The optimized MLR extract was evaluated as an antioxidant for the preservation of raw salmon. The samples were divided into the following groups:

- Group 1—oxidized salmon: 2 g of salmon + 0.5 mL of ozonized water + 0.5 mL of water.
- Group 2—oxidized salmon + optimized MLR extract: 2 g of salmon + 0.5 mL of ozonized water + 0.5 mL of optimized MLR extract.
- Group 3—oxidized salmon + BHT 200 ppm: 2 g of salmon + 0.5 mL of ozonized water + 0.5 mL of BHT.
- Group 4—oxidized salmon + BHA 200 ppm: 2 g of salmon + 0.5 mL of ozonized water + 0.5 mL of BHA.
- Group 5—control salmon: 2 g of salmon + 1 mL of water.

Salmon was stored at 4 °C and was analyzed using the 2-TBA reactive substance assay on days 0, 1, 4, 7, and 14. Additionally, salmon oxidation tests were conducted using heat treatment at 95 °C for 40 min (Figure S3). For comparison purposes, one group consisted of cooked salmon (2 g of salmon + 1 mL of water), and another group was cooked salmon with optimized MLR extract (2 g of salmon + 0.5 mL of water + 0.5 mL of optimized MLR extract).

#### 2.9.3. 2-Thiobarbituric Acid Reactive Substances (TBAR) Assay

The procedure reported by Albishi et al. [17] was followed to determine the malondialdehyde content as a secondary product of oxidation in salmon. Briefly, 1 g of raw salmon samples was mixed with 2.5 mL of TCA (10% *w*/*v*) and vortexed for 2 min. Then, it was mixed with 2.5 mL of aqueous TBA solution (0.02 M), vortexed for 30 s, and centrifuged at 3000× *g* for 10 min. The supernatant was recovered and heated in boiling water for 45 min. Finally, the sample was cooled, and absorbance was measured at 532 nm using a spectrophotometer (N4S UV-VIS, Hinotek, Ningbo, Zhejiang, China). A standard curve of malondialdehyde (MDA) in the concentration range of 1–10 ppm was prepared using 1,1,3,3-Tetraethoxypropane as an MDA precursor. The TBAR values were expressed as MDA equivalents per kilogram of salmon (mg MDA eq/kg).

### 2.10. Statistical Analysis

Design-Expert software version 12 was used for the optimization design. Stata version 15 was used to perform analysis of variance (ANOVA), followed by the use of Tukey’s post hoc test to compare the means of the phenolic profile according to the different extraction methods evaluated and TBAR analysis during salmon storage. A significance level of 0.05 was applied in all cases.

## 3. Results

### 3.1. Total Phenolic Content in Maqui Leaves and Maqui Leaf Waste

TPC was used to determine, at the laboratory level, the phenolic content remaining in maqui leaf waste after the infusion process. The maqui leaves had an initial TPC of 65.54 mg GAE/g, and after the infusion process, MLR retained 45.44 mg GAE/g, representing 69.33% of the original TPC. These results show that MLR recovered after the infusion process is still a significant source of polyphenols.

### 3.2. Optimization of Polyphenol Extraction with Ultrasound

Results of the ultrasound optimization are shown in Table 1. A second-degree polynomial model was fitted to the results with high R^2^ and adjusted R^2^ values for total phenolic content (0.9432 and 0.8935), FRAP antioxidant capacity (0.9417 and 0.9029), and ORAC antioxidant capacity (0.8175 and 0.7263). The polynomial model is shown in Equations (1)–(3).

TPC = −50.42 + 1.41 × Time + 3.00 × MLR:Water + 0.98 × Power − 0.02 × Time × MLR:Water − 0.03 × Time^2^ − 0.04 × MP:Agua^2^ − 0.01 × Power^2^ (1)

FRAP = −523.07 + 10.64 × Time + 33.28 × MLR:Water + 10.21 × Power − 0.27 × Time^2^ − 0.60 × MLR:water^2^ − 0.07 × Power^2^ (2)

ORAC = −11.70 − 0.11 × Time + 1.85 × MLR:Water + 0.53 × Power − 0.03 × MLR:Water^2^ + 0.003 × Power^2^ (3)

**Figure 1:**
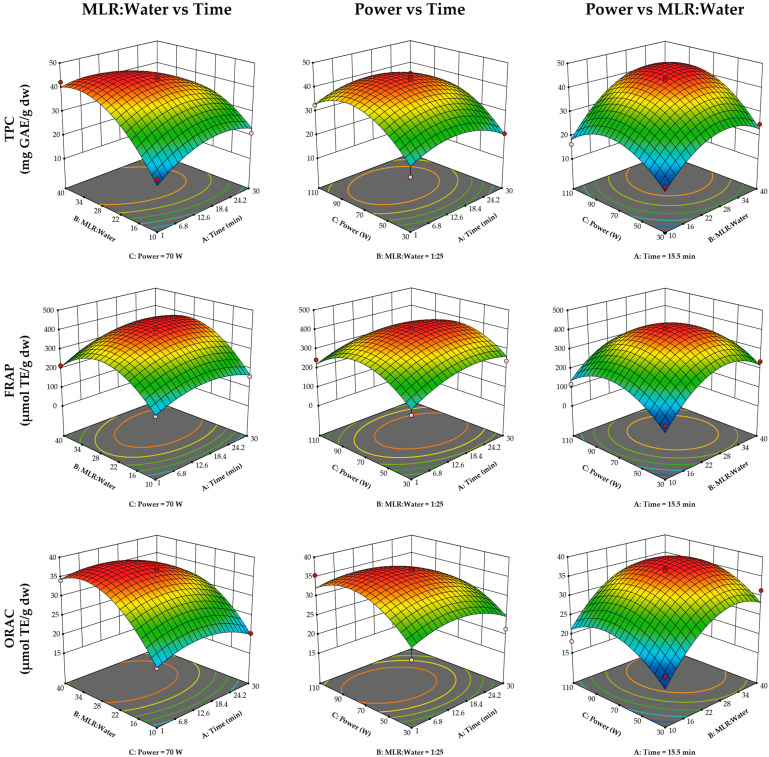
Response surface plots for ultrasound-assisted extraction of phenolics from maqui leaf residue. MLR: maqui leaves residue; TPC: total phenolic content; GAE: gallic acid equivalent; FRAP: ferric reducing antioxidant potential; ORAC: oxygen radical absorbance capacity; TE: trolox equivalent; dw: dry weight.

The response surface plots are shown in Figure 1. The MLR–water ratio was the most influential variable in the ultrasound extraction process (Table 2), with the lowest TPC and with FRAP and ORAC antioxidant capacities observed at the lowest ratio (1:10). Ultrasound power significantly influenced TPC, reaching its maximum at approximately 80 W, but decreasing as it approached 110 W. In contrast, ultrasound power had no significant effect on antioxidant capacity. Similarly, extraction time did not have a significant effect on any of the response variables.

The optimized ultrasound extraction conditions for achieving maximum TPC and antioxidant capacity (FRAP and ORAC) were defined as 6 min, MLR–water ratio = 1:30, and 70 W power (desirability function = 0.934). Experimental results obtained under these conditions are shown in Table 1, and these were similar to the predicted model values for TPC (43.14 mg GAE/g dry weight), FRAP (359.15 µmol TE/g dry weight), and ORAC (36.81 µmol TE/g dry weight).

### 3.3. Phenolic Profile (UPLC-ESI-MS/MS)

The MLR extract obtained by ultrasound-assisted extraction was characterized for its phenolic profile and compared with the profiles of two conventional extraction methods, one using water and the other a solvent mixture (methanol–water–acetone, 3:4:3 *v*/*v*/*v*) (Table 3). Both phenolic acids and flavonoids were detected, with flavonoids being the most abundant compounds.

Up to eight different phenolic acids were detected in MLR. The total phenolic acid content was highest in the extract obtained by ultrasound, followed by the extract obtained with the organic solvent mixture, and the lowest was seen in the extract obtained with water. The most abundant phenolic acids in the MLR extracts were gallic acid, chlorogenic acid, and cryptochlorogenic acid. Gallic acid was the most prevalent in the extracts obtained by ultrasound and water methods, with concentrations of 158.48 and 52.47 µg/g, respectively. In contrast, chlorogenic acid was the most abundant in the MLR extract obtained using the organic solvent mixture.

Likewise, up to eight different flavonoids were identified in MLR. The total flavonoid content, similar to that of phenolic acids, was highest in the extract obtained by ultrasound-assisted extraction compared to the extraction controls (water and methanol/acetone/water). Luteolin was the most abundant flavonoid in MLR under ultrasound extraction, with a content of 293.06 µg/g. In contrast, rutin was the dominant flavonoid in the extracts obtained using water and methanol/acetone/water, with concentrations of 321.72 and 237.81 µg/g, respectively, compared to a concentration of 54.78 µg/g in the extract obtained by ultrasound.

Ultrasound extraction recovered a greater amount of phenolics compared to extractions with solvent mixtures (methanol–water–acetone, 3:4:3 *v*/*v*/*v*) and water. The phenolic profile in the MLR extract obtained by ultrasound showed higher contents of gallic, caffeic, cryptochlorogenic, vanillic acids, luteolin, kaempferol, epicatechin, and quercitrin compared to the control extractions (*p* < 0.05). The results highlight the use of ultrasound as an effective technology for recovering a greater quantity of polyphenols. However, catechin was not extracted through ultrasound, and lower concentrations of rutin and quercetin were obtained compared to the solvent mixture extraction.

### 3.4. Effect of Maqui Leaf Residue Extract on Salmon Oxidation

**Figure 2:**
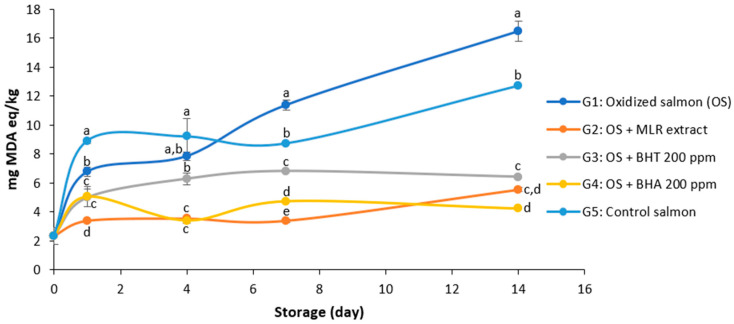
Protective effect of maqui leaf residue (MLR) on ozone-induced oxidation in a fish model system during storage 4 °C as evaluated by the 2-thiobarbituric acid reactive substance (TBAR) assay. MDA: malondialdehyde; G: group, MLR (916.65 µg/g). BHA: butylated hydroxyanisole; (BHT): 3,5-Di-tert-4butylhydroxytoluene (BHT). Values are expressed as mean ± standard deviation (n = 3). Different letters in the same day indicate significant differences according to Tukey’s test (*p* < 0.05).

Figure 2 shows the malondialdehyde (MDA) levels over the storage period of salmon under different treatment groups. The optimized MLR extract obtained by ultrasound, containing 916.65 µg polyphenols/g dw (Table 3), was used to evaluate its effectiveness in preserving salmon. Ozonized water (37 mg/mL) increased MDA formation in the salmon, reaching a maximum value of 16.49 mg MDA eq/kg on day 14. MDA formation was significantly reduced by the synthetic antioxidants BHT and BHA, and by the optimized MLR extract, decreasing MDA levels by 61.09%, 74.22%, and 66.44%, respectively, on day 14. At the end of the storage period, no significant differences in MDA levels were observed between the 200 ppm concentration of the synthetic antioxidant BHT and the optimized MLR extract, nor between the MLR extract and the 200 ppm concentration of BHA. To assess whether ozonated water induced oxidation in salmon, a control group was included in which salmon was stored with water alone (Group 5). This group exhibited a significantly lower increase in MDA levels compared to those stored with ozonated water.

## 4. Discussion

In comparison to the maqui leaves analyzed in this study, similar total phenolic content (TPC) values have been reported, such as the 69 mg GAE/g result reported by Rubilar et al. [20] for maqui leaves from the Araucanía region in Chile. Higher TPC values have been reported in maqui leaves from the same region, such as 264.53 mg GAE/g [21] and 148.76 mg GAE/g [28]. The variation in TPC values in maqui leaves depends on factors such as the planting area, management practices, processing methods, and climate [29]. Concerning leaf residues, Tsubaki et al. [30] reported that the residue of green, oolong, and black tea leaves still contained 12.7%, 18.7%, and 23.3% of total polyphenols, respectively. A similar trend was observed by Abdeltaif et al. [31] in black tea leaves, reporting 187.50 mg GAE/g and 152.87 mg GAE/g in the leaf waste after brewing tea, representing 81.53% of the initial TPC value. Variation in TPC in residual leaves depends on the infusion process they undergo, including parameters such as extraction time and temperature [31].

TPC values increase with a higher MLR–water ratio (Table 1). Similar results were reported by Jovanović et al. [32] for *Thymus serpyllum* L. extracts, where lower TPC values were obtained with a 1:10 ratio, possibly due to increased viscosity with a higher amount of plant material, which interfered with the propagation of ultrasonic waves in the extract. As the solvent amount increased (greater MLR:Water ratio) up to 1:30, the amount of extracted TPC also increased. This trend, where more solvent improves the extraction of TPC, has also been reported in blackberry leaves (*Morus nigra* L.) [33].

Increasing extraction power (Figure 1) favored the extraction of phenolics, as higher power promotes the rupture of cell walls, allowing the solvent to penetrate the plant matrix and increase the diffusion coefficient [34]. While some authors report this increase, such as Abi-Khattar et al. [35], who evaluated a range of 100 to 400 W and found greater TPC recovery in olive leaves as extraction power increased, in the case of MLR, a decrease in TPC was observed when power reached 110 W during ultrasound extraction. This decrease may have been due to the production of free radicals during ultrasonic cavitation, which potentially accelerated the oxidation of some phenolic compounds. This effect was reported by Ji et al. [36], who observed an increase in phenol extraction in coffee leaves up to 120 W; however, at 150 and 180 W, the amount of extracted phenolics decreased.

While increasing extraction time did not produce significant differences in TPC yield (Table 2), the interaction of time, power, and MLR–water ratio for maximum phenolic extraction and antioxidant capacity resulted in an optimal extraction time of 6 min, which represents an advantage over other reported extractions. Rivera-Tovar et al. [21] reported an extraction time of 1 h for phenolics from maqui leaves when using 50% methanol (pH 2), followed by 70% acetone. This time was reduced to 15 min when the authors used pressurized liquids, another emerging technology for polyphenol recovery. In contrast, Flores et al. [37] reported 48 h of extraction, using a methanol and ethyl ether mixture to obtain phenolic extracts from maqui leaves. These findings highlight the need to optimize the phenolic extraction process and the importance of using ultrasound technology, which is efficient in a shorter time and only requires water as a solvent, compared to methods that rely on organic solvents like those mentioned earlier.

Although there are no reports about the phenolic profile of MLR, Vidal et al. [38] reported gallic acid as the most abundant phenolic acid present in maqui leaves from the Bio Bio region of Chile. In addition, Rivera-Tovar et al. [21] reported chlorogenic acid as the phenolic acid with the highest concentration (1440 µg/g) in maqui leaves from the La Araucanía region of Chile, followed by gallic acid (640 µg/g). The differences in polyphenol content may be attributed to the geographical origin of the maqui leaves.

Luteolin was the most abundant flavonoid present in the MLR extract obtained through ultrasound-assisted extraction. This flavonoid has been reported in plants used in traditional medicine and is of interest for its potential pharmacological properties, such as anti-inflammatory, antioxidant, and neuroprotective effects [39]. Luteolin content has been reported to be 2258 µg/g in perilla leaves (Perilla frutescens L.) [40], 170 µg/g in moringa leaves (*Moringa oleifera*) [41], and 3050 µg/g in kaffir lime leaves (*Citrus hystrix*) [42]. Flavonoids such as quercetin, isoquercitrin, hyperoside, quercitrin, rutin, and phenolic acids have been reported in maqui leaves from the Araucanía and Bio Bio regions [20,21,38]. However, this is the first study to identify the presence of luteolin in MLR.

Ultrasound creates cavitation bubbles in plant cells, which break the cell walls and facilitate the release of phenolic compounds [43]. Algan Cavuldak et al. [33] reported an improvement in phenolic extraction from 18.28 to 21.78 mg GAE/g in black mulberry leaves (*Morus nigra* L.) when ultrasound was used compared to water extraction. This may explain the higher phenolic content extracted by ultrasound. However, in the present study the rutin content was significantly lower in the extract obtained by ultrasound compared to the extraction controls, and the quercetin content in the ultrasound-derived extract was significantly lower compared to the one obtained with the organic solvent mixture. This could be due to the hydrolysis of rutin under the ultrasound conditions used (70 W, 6 min), as rutin is composed of quercetin linked to the disaccharide rutinose, which consequently reduces the rutin content. Qiao et al. [44] studied the stability of 14 flavonoids, finding that quercetin experienced greater degradation under ultrasound intensities of 1529 to 2395 W/cm^2^. On the other hand, Wang et al. [45] reported that after 45 min of ultrasound at 120 W, the rutin content decreased by 78.9% due to hydroxyl radical formation. This suggests that the degradation is not limited to quercetin and rutin, but could potentially affect other flavonoids, such as catechin, which was not detected in the ultrasound extract but was present in the control extractions. Additionally, it has been described how several parameters, such as the type of solvent, temperature, liquid height, ultrasound intensity, pulse length, and duty cycle, can influence the extent of degradation under ultrasound conditions [44].

The effectiveness of natural antioxidants has been tested in salmon models, where lipid oxidation is evaluated during the storage of cooked salmon [16], smoked salmon [17] or raw salmon exposed to gamma irradiation-induced oxidation [18]. Salmon is a source of polyunsaturated fatty acids that are sensitive to oxidation, making it a useful model for studying and monitoring lipid oxidation [17]. Other methods to induce lipid oxidation have been reported, such as gamma irradiation. de Camargo et al. [18] used irradiation at 3.0 kGy on raw salmon samples, salmon with peanut by-product extract, and salmon with 200 ppm of BHA, obtaining lower MDA values during storage at 4 °C for 7 days in the salmon with peanut by-product extract and 200 ppm of BHA. On the other hand, Albishi et al. [16] induced lipid oxidation by heating salmon samples containing potato extracts at 80 °C for 40 min, which inhibited TBAR formation up to 83.4% after 7 days of storage. Similar results were observed with cooked salmon samples heated at 95 °C for 40 min with optimized MLR extract, reaching a value of 8.22 mg MDA eq/kg in cooked samples after 14 days of storage at 4 °C (Figure S3).

Tanaka et al. [14] reported that treatment with ozonated water can generate reactive oxygen species, accelerating protein denaturation and lipid oxidation. This effect is demonstrated by the increase in TBAR values in the salmon samples (Figure 2). When the optimized MLR extract (916.65 µg/g) was added, the MDA value was reduced by 76.03% compared to the cooked salmon (Figure S3). Other extracts with similar trends in reducing lipid oxidation have been reported, as occurs with the use of a phenolic extract from olive vegetation water on fresh salmon steak [19], the use of stevia extracts during refrigerated salmon paste preservation [46], and the application of pomegranate peel extract to cooked meat [47]. However, to the best of our knowledge, this is the first time that a phenolic extract has been tested using ozone as the oxidizing agent. In summary, the MLR extract prevented the ozone-induced lipid oxidation of salmon, minimizing the effects of this process and presenting itself as a natural antioxidant alternative.

## 5. Conclusions

The ultrasound-assisted extraction of phenolic compounds from maqui leaf residue was optimized, resulting in a short and sustainable extraction process to recover higher levels of polyphenols compared to conventional methods. The extracted polyphenols demonstrated significant antioxidant activity, which was confirmed by their capacity to inhibit ozone-induced oxidation in a fish model system. The results suggest that residues generated upon infusion processing could be valorized as a natural source of antioxidants for food preservation, offering a sustainable and functional solution.

## Figures and Tables

**Table 1:** Total phenolic content and antioxidant capacity of maqui leaf residue using the Box–Behnken design.

Experiment	Time (min)	MLR:Water	Power (W)	TPC (mg GAE/g dw)	FRAP (µmol TE/g dw)	ORAC (µmol TE/g dw)
1	1	1:10	70	17.63	144.82	21.18
2	30	1:10	70	20.98	158.99	20.34
3	1	1:40	70	42.26	216.97	34.09
4	30	1:40	70	26.12	319.63	29.42
5	1	1:25	30	18.42	149.56	23.15
6	30	1:25	30	20.65	240.02	21.47
7	1	1:25	110	32.72	245.64	35.44
8	30	1:25	110	35.24	287.39	29.87
9	15.5	1:10	30	14.88	99.10	19.11
10	15.5	1:40	30	24.95	238.04	31.49
11	15.5	1:10	110	16.30	118.27	18.11
12	15.5	1:40	110	37.15	160.67	28.50
13	15.5	1:25	70	41.12	401.34	36.95
14	15.5	1:25	70	42.15	400.72	36.59
15	15.5	1:25	70	44.12	405.39	35.75
16	15.5	1:25	70	43.14	400.24	36.19
17 *	6	1:30	70	44.26	301.21	41.64

* optimized conditions—MLR: maqui leaves residue; TPC: total phenolic content; GAE: gallic acid equivalent; FRAP: ferric reducing antioxidant potential; ORAC: oxygen radical absorbance capacity; TE: Trolox equivalent; dw: dry weight.

**Table 2:** Analysis of variance for a quadratic model of TPC, FRAP, and ORAC.

Source	TPC			FRAP			ORAC		
	Sum of Squares	df	*p*-Value	Sum of Squares	df	*p*-Value	Sum of Squares	df	*p*-Value
Model	1719.31	9	0.0016	172,001.03	9	0.0007	656.46	9	0.0236
A: Time	8.08	1	0.4456	7752.01	1	0.0267	20.34	1	0.2559
B: MLR:Water	460.38	1	0.0008	21,437.16	1	0.0028	250.59	1	0.0045
C: Power	225.91	1	0.005	908.65	1	0.3562	34.86	1	0.1513
AB	94.98	1	0.0312	1957.15	1	0.1928	3.67	1	0.6128
AC	0.021	1	0.9682	593.23	1	0.4502	3.78	1	0.6077
BC	29.05	1	0.1726	2329.89	1	0.1607	1	1	0.7902
A^2^	154.95	1	0.0117	13,268.4	1	0.0088	48.04	1	0.1019
B^2^	373.37	1	0.0014	72,064.74	1	0.0001	176.67	1	0.0101
C^2^	372.57	1	0.0015	51,689.81	1	0.0003	117.51	1	0.0235
Residual	72.74	6		5459.16	6		77.41	6	
Lack of fit	67.75	3	0.0299	5442.52	3	0.0003	76.6	3	0.0018
Pure error	4.99	3		16.64	3		0.8	3	
Cor Total	1792.05	15		177,460.19	15		733.87	15	

MLR: maqui leaf residue; TPC: total phenolic content; FRAP: ferric reducing antioxidant potential; ORAC: oxygen radical absorbance capacity; df: degrees of freedom.

**Table 3:** Polyphenols (µg/g dw) in the soluble fraction of maqui leaf residue obtained by different extraction methods.

Polyphenols	Ultrasound	Water	Methanol–Water–Acetone (3:4:3, *v*:*v*:*v*)
Phenolic acids			
Gallic acid	158.48 ± 10.78 ^b,A^	52.47 ± 2.09 ^b,C^	99.43 ± 3.26 ^c,B^
Syringic acid	nd	0.04 ± 0.00 ^h^	nd
Ferulic acid	tr	tr	tr
Chlorogenic acid	105.70 ± 1.54 ^d,B^	43.72 ± 0.65 ^c,C^	135.16 ± 3.37 ^b,A^
Caffeic acid	2.35 ± 0.58 ^g,A^	tr	0.45 ± 0.01 ^h,B^
p-Coumaric acid	tr	0.36 ± 0.00 ^h,A^	0.20 ± 0.00 ^h,B^
Cryptochlorogenic acid ^1^	111.57 ± 14,34 ^d,A^	23.16 ± 0.66 ^e,C^	42.06 ± 0.39 ^f,B^
3,4-Dihydroxybenzoic acid ^2^	3.50 ± 0.92 ^g,C^	5.74 ± 0.42 ^f,g,B^	7.15 ± 0.16 ^g,h,A^
Vanillic acid ^2^	0.58 ± 0.11 ^g^	nd	nd
Total phenolic acids	382.18	125.49	284.45
Flavonoids			
Catechin	nd	3.20 ± 0.06 ^f,g,h,B^	4.88 ± 0.14 ^h,A^
Rutin	54.78 ± 1.89 ^e,C^	321.72 ± 2.87 ^a,A^	237.81 ± 7.33 ^a,B^
Quercetin	17.92 ± 0.06 ^f.g.B^	6.37 ± 0.41 ^f.C^	49.85 ± 0.87 ^e.A^
Luteolin	293.06 ± 3.70 ^a.A^	53.61 ± 1.90 ^b.B^	60.68 ± 0.43 ^d.B^
Kaempferol	137.18 ± 6.56 ^c,A^	32.64 ± 1.62 ^d,C^	51.91 ± 1.40 ^e,B^
Epicatechin	nd	2.28 ± 0.04 ^g,h,A^	2.88 ± 0.06 ^h,A^
Myricetin	nd	nd	nd
Isorhamnetin	tr	5.97 ± 0.30 ^f,B^	13.65 ± 0.26 ^g,A^
Quercitrin ^3^	31.46 ± 2.47 ^f,A^	6.22 ± 0.04 ^f,C^	12.71 ± 0.06 ^g,B^
Total flavonoids	534.4	432.01	434.37
Total polyphenols	916.65	557.51	718.83

^1^ mg chlorogenic acid equivalent/g, ^2^ mg gallic acid equivalent/g, ^3^ mg catechin equivalent/g. ms: dry weight; nd: not detected; tr: traces. Values are expressed as mean ± standard deviation (n = 3). Different capital letters in the same row indicate significant differences according to Tukey’s test (*p* < 0.05). Different lowercase letters in the same column indicate significant difference according to Tukey’s test (*p* < 0.05).

## Data Availability

Data are available on request from the authors.

## Supplementary Materials

The following supporting information can be downloaded at: https://www.mdpi.com/article/10.3390/antiox14030263/s1, Table S1: Mixture design for optimizing the solvent composition in polyphenol extraction from maqui leaf residue; Table S2: Detection and quantification limits and R^2^ for the quantification of polyphenols by UPLC-ESI-MS/MS; Figure S1: Response surface plot for optimizing the solvent composition in polyphenol extraction from maqui leaf residue; Figure S2: Instrument setup for ultrasound extraction; Figure S3: Storage of salmon treated with heat treatment (95 °C for 40 min) and optimized MLR extract.
