# Supplementary material for: Phenolics of Maqui Leaf Residues Exhibit Antioxidant Properties Against Ozone-Induced Oxidation in Fish Model Systems
Source: Antioxidants (Basel). 2025 Feb 26;14(3):263. doi: 10.3390/antiox14030263 (PMC11939210; doi:10.3390/antiox14030263)
Supplement: Supplementary file 1 [file antioxidants-14-00263-s001.zip › antioxidants-3482923-supplementary.pdf]

Table S1. Mixture design for optimizing the solvent composition in polyphenol extraction from maqui leaf residue

Experiment	Solvent mixture			TPC
	Acetone	Water	Methanol	(mg GAE/g dw)
1	100.00	0.00	0.00	2.08
2	0.00	100.00	0.00	19.30
3	0.00	0.00	100.00	25.34
4	50.00	50.00	0.00	30.80
5	50.00	0.00	50.00	14.46
6	0.00	50.00	50.00	29.57
7	33.33	33.33	33.33	45.17
8	33.33	33.33	33.33	44.81
9	33.33	33.33	33.33	42.13
10	33.33	33.33	33.33	41.35
11*	30.0	40.0	30.0	45.57

* Optimized conditions, TPC: Total phenolic compounds, GAE: Gallic acid equivalent, dw: dry weight

Table S2. Detection and quantification limits and R^2 for the quantification of polyphenols by UPLC-ESI-MS/MS

Polyphenols	Structure	LOD (μg/L)	LOQ (μg/L)	R^2
**Phenolic acids**				
Gallic acid	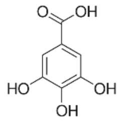	41	124	0.9988
Syringic acid	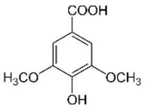	55	167	0.9995
Ferulic acid	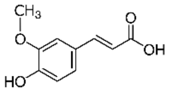	110	334	0.9944
Chlorogenic acid	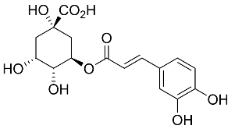	51	154	0.9994
Caffeic acid	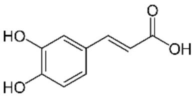	142	430	0.9976
p-Coumaric acid	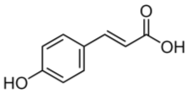	124	377	0.9911
**Flavonoids**				
Catechin	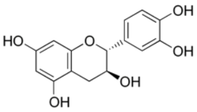	49	150	0.9997

Rutin	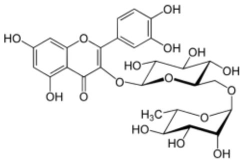	249	756	0.9939
Quercetin	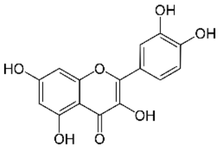	67	203	0.9969
Luteolin	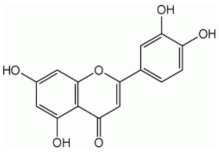	136	412	0.9965
Kaempferol	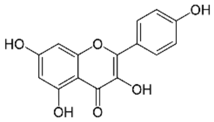	390	1181	0.9905
Epicatechin	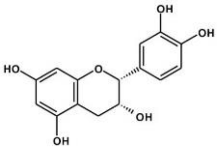	83	252	0.9991
Myricetin	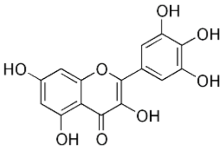	180	545	0.9956
Isorhamnetin	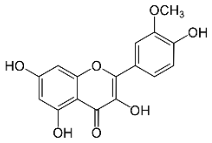	198	756	0.9939

LOD: Limit of detection, LOQ: Limit of quantification

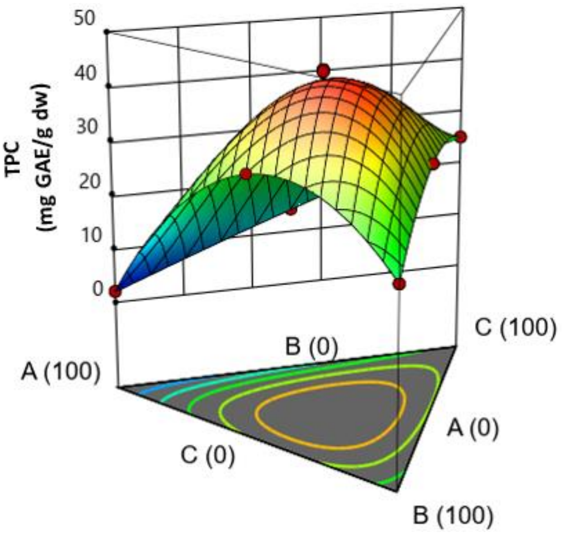

Figure S1. Response surface plot for optimizing the solvent composition in polyphenol extraction from maqui leaf residue. TPC: Total phenolic compounds, GAE: Gallic acid equivalent, dw: dry weight, A: Acetone, B: Water, C: Methanol

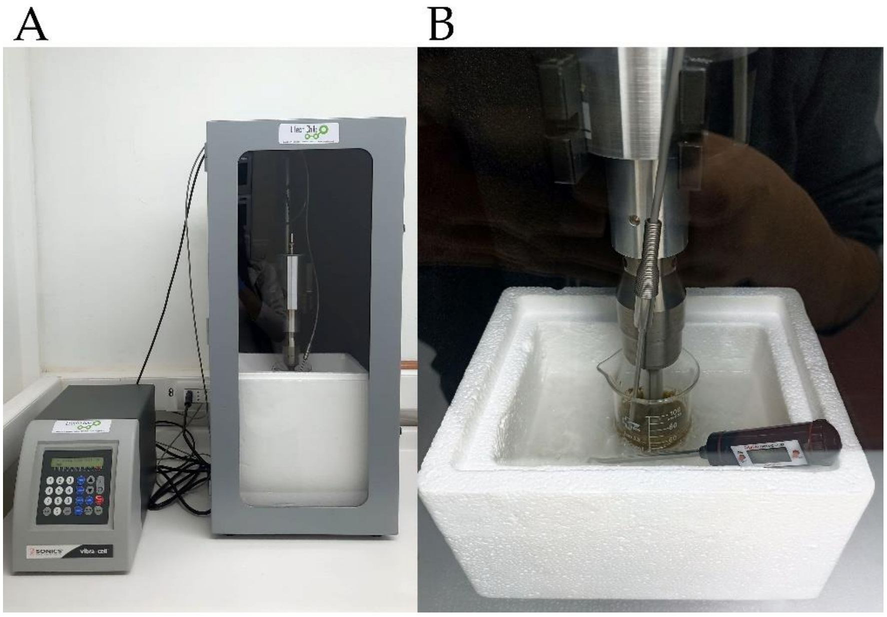

Figure S2. Instrument setup for ultrasound extraction. **(A)** Ultrasonic homogenizer (Sonic VCX 750), **(B)** Sample placed in an ice-water bath to control temperature during extraction

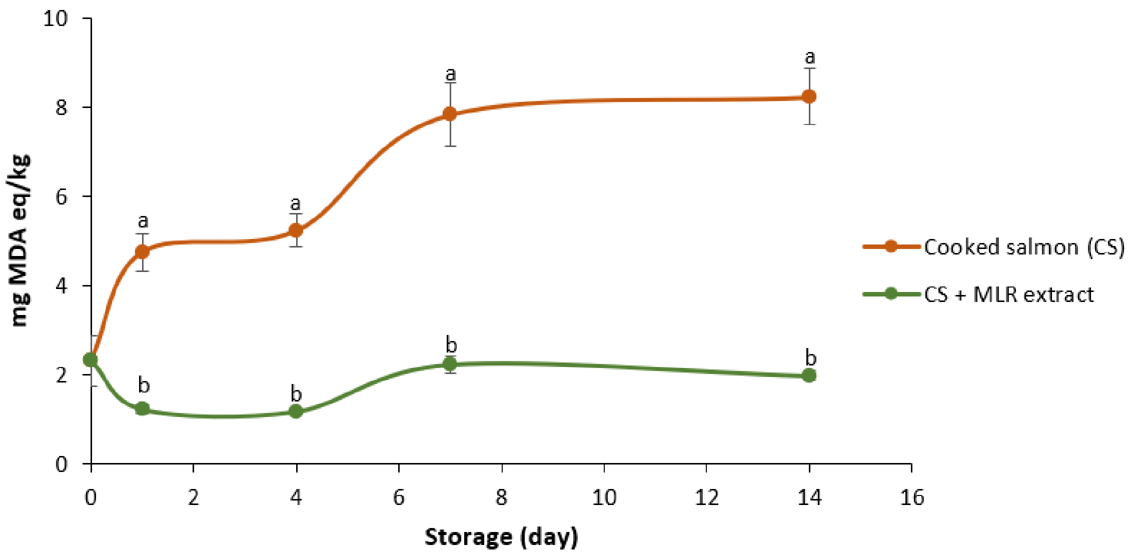

Figure S3. Storage of salmon treated with heat treatment (95 °C for 40 min) and optimized MLR extract. MLR: Maqui leaf residue (916.65 μg/g). Values are expressed as mean ± standard deviation ($n = 3$). Different letters in the same day indicate significant difference according to T-test ($p < 0.05$)
